# Acute Immunocompatibility Profiling of Linear Poly(FTC) for Long-Acting Emtricitabine Delivery

**DOI:** 10.3390/pharmaceutics18101296

**Published:** 2026-10-13

**Authors:** Danielle E. Brain, Faye Y. Hern, Anika Shakil, Chung Liu, Caren Freel Meyers, Steve P. Rannard, Andrew Owen, Neill J. Liptrott

**Affiliations:** 1 Immunocompatibility Group, Materials Innovation Factory, University of Liverpool, Liverpool L7 3NY, UK; danielle.brain@liverpool.ac.uk; 2 Centre of Excellence for Long-Acting Therapeutics, William Henry Duncan Building, University of Liverpool, Liverpool L69 7ZA, UK; 3 Department of Pharmacology and Molecular Sciences, The Johns Hopkins University School of Medicine, Baltimore, MD 21287, USA

**Keywords:** polymer of prodrug (POP), long-acting therapeutics (LATs), immunocompatibility, complement

## Abstract

**Background/Objectives**: Long-acting therapeutics are being investigated for their potential to enhance medication adherence and improve overall treatment outcomes. Due to the prolonged residence time at the administration site, such as subcutaneous insertion, long-acting preparations may modulate the responses of local immune cells linked to material characteristics. Long-acting Polymer of Prodrug (POP) of Nucleoside Reverse Transcriptase Inhibitor (NRTI) Emtricitabine (FTC) has been developed for subcutaneous administration. **Methods**: The present study focused on the acute immunocompatibility of an FTC-based POP and its interactions with various cells and components of the immune system following acute exposure using DMSO-dispersed polymer linear poly(FTC). A tiered assessment was carried out, including assessment of pyrogens (MAT), cytotoxicity (MTT/LDH), Leukocyte proliferation (MTT), complement proteins (iC3b, Factor H, sC5b-9, Bb and C4d EIAs) and cytokine secretion in whole blood (Luminex panel). **Results**: Linear poly(FTC) had no detectable pyrogenic activity, no overt cytotoxicity within the THP-1 cell line, no consistent leukocyte proliferative activation and no broad whole-blood cytokine induction. Complement analysis, however, indicated a concentration-associated higher iC3b signal by linear poly(FTC) and a higher Bb signal by linear poly(FTC) at 80 µg/mL when compared to vehicle (0.4% DMSO). **Conclusions**: Further investigations are warranted to assess the long-term implications of these implants, given their extended residence times. Therefore, the adaptation of existing safety assessments should be prioritized to facilitate the safe translation of these POPs into clinical studies.

## 1. Introduction

Long-acting therapeutics (LATs) already improve the treatment of several conditions and hold promise for many additional applications [1,2]. Any new therapeutic requires assessment for possible safety issues, particularly LATs, with respect to the immune system due to their physicochemical characteristics and local immune activation at the site of administration [3,4,5,6]. Early assessment of the acute biocompatibility of any new material in development is crucial to evaluate its potential to cause problems in humans. Immunocompatibility assessments are also essential early in development due to previous local toxicity associated with the Tenofovir Alafenamide (TAF) non-biodegradable implants tested in vivo [7]. We have previously demonstrated how the highly water-soluble FTC can be used to create a polymer prodrug that can release FTC in plasma [8].

These POP preparations are designed as fully biodegradable implants that do not require an implant system. There is a need for long-acting delivery of ARVs, particularly NRTIs, due to their water solubility. One POP material containing the drug FTC was assessed in this paper. Bioburden assessment is standard practice prior to any immunological evaluation, particularly for materials not manufactured to good manufacturing practice (GMP) standards [9]. Following the bioburden assessment, the materials are evaluated for their cytotoxicity to determine concentrations for subsequent assays and ensure that subsequent assessment is not influenced by cytotoxicity [10]. Common acute immunotoxicity assessments must then be carried out to highlight materials that have the potential to cause major safety issues in humans and ensure that these materials are safe. Standardised in vitro immunocompatibility workflows have been developed to identify early material-associated liabilities, including pyrogenicity, cytotoxicity, leukocyte activation, complement activation and cytokine release. If any significant adverse results are observed in these assays, they are subsequently investigated to identify the pathway and mechanism responsible and to assess whether these issues are detrimental to the disease or treatment for which the materials will be used [11]. Common acute immunotoxicity assays encompass a variety of immunological systems, including, but not limited to, leukocyte proliferation and complement.

Lymphocytes circulate in the blood and provide protection against infections. The proliferation of lymphocytes can indicate immune activation due to material exposure and is part of material screening for immunological safety. Complement is a crucial component of the innate immune system, responding to danger signals, resulting in the release of anaphylatoxins and the formation of the membrane attack complex (MAC) [12]. Complement activation has been observed in several currently used nanoformulated drugs; consequently, investigating complement in the preclinical assessment of new long-acting delivery systems is vital [13,14].

Exploring whole-blood responses to new materials can be important for determining their safety, particularly if they are expected or have the potential to enter the bloodstream. This paper explores the acute immunocompatibility of an FTC-containing POP preparation and its interactions with cells and components of the immune system following acute exposure. This work aimed to explore the acute immune interaction of these newly developed POP implants through a tiered workflow to assess their acute safety in humans.

## 2. Materials and Methods

### 2.1. Materials

FTC was purchased from Wischem (Shanghai, China). Phytohaemagglutinin-M (PHA-M) (Sigma Aldrich, Gillingham, UK), Thiazolyl Blue Tetrazolium Bromide (MTT) powder (Sigma Aldrich), Dimethyl sulfoxide (DMSO) (Sigma Aldrich), PyroMAT^®^ kit (Millipore, Merck, Gilligham, UK), Roswell Park Memorial Institute (RPMI)-1640 (Sigma Aldrich), Phosphate Buffered Solution (PBS) (Sigma Aldrich), Cytotoxicity Detection Kit^PLUS^ Lactate dehydrogenase (LDH) (Roche) (Darmstadt, Germany). Ficoll-paque was purchased from Fisher Scientific (Loughborough, UK). MicroVue™ Complement iC3b/Bb/C4d/Factor H/sC5b-9 Enzyme Immunoassays (EIAs), Cobra venom factor (CVF) were all purchased from Quidel (San Diego, CA, USA). Doxil (Dox-NP^®^) was purchased from Avanti Polar Lipids (Alabaster, AL, USA). LPS-EK Ultrapure, oligodeoxynucleotide (ODN) 2006, 2′3′-cyclic guanosine monophosphate-adenosine monophosphate (cGAMP), Poly(dG:dC)/LyoVec™, Herpes Simplex virus (HSV)-60/LyoVec™ were all purchased from Invivogen (Toulouse, France). 9-plex human Luminex assay (cytokines: Interferon (IFN)-α, IFN-β, IFN-γ, IL-1b, IL-1Receptor Antagonist (RA), IL-6, IL-8, IL-18, and Tumour Necrosis Factor (TNF)-α, were purchased from R&D Systems, BioTechne (Minneapolis, MN, USA). Bio-plex 200 system and Sheath fluid were both purchased from Bio-rad (Hercules, CA, USA). Foetal Bovine Serum (FBS) (Gibco) was purchased from ThermoFisher Scientific (Waltham, MA, USA). The THP-1 cell lines were sourced from ATCC, purchased from LGC (Guildford, UK). The CLARIOstar plate reader was purchased from BMG Labtech (Ortenberg, Germany). GraphPad Prism software 10.2.3 (Boston, MA, USA).

Gemini Notebook (v. 1.47.6.944047133) was used as an AI-assisted visualisation tool during the preparation of the graphical abstract. The authors provided original prompts derived from this study’s aims, experimental approach, and principal conclusions. The authors critically reviewed the resulting graphical output, modified it where necessary, and approved it. All scientific interpretation, figure content, and final presentation remain the responsibility of the authors.

### 2.2. POP Material Preparation

**Figure 1:**
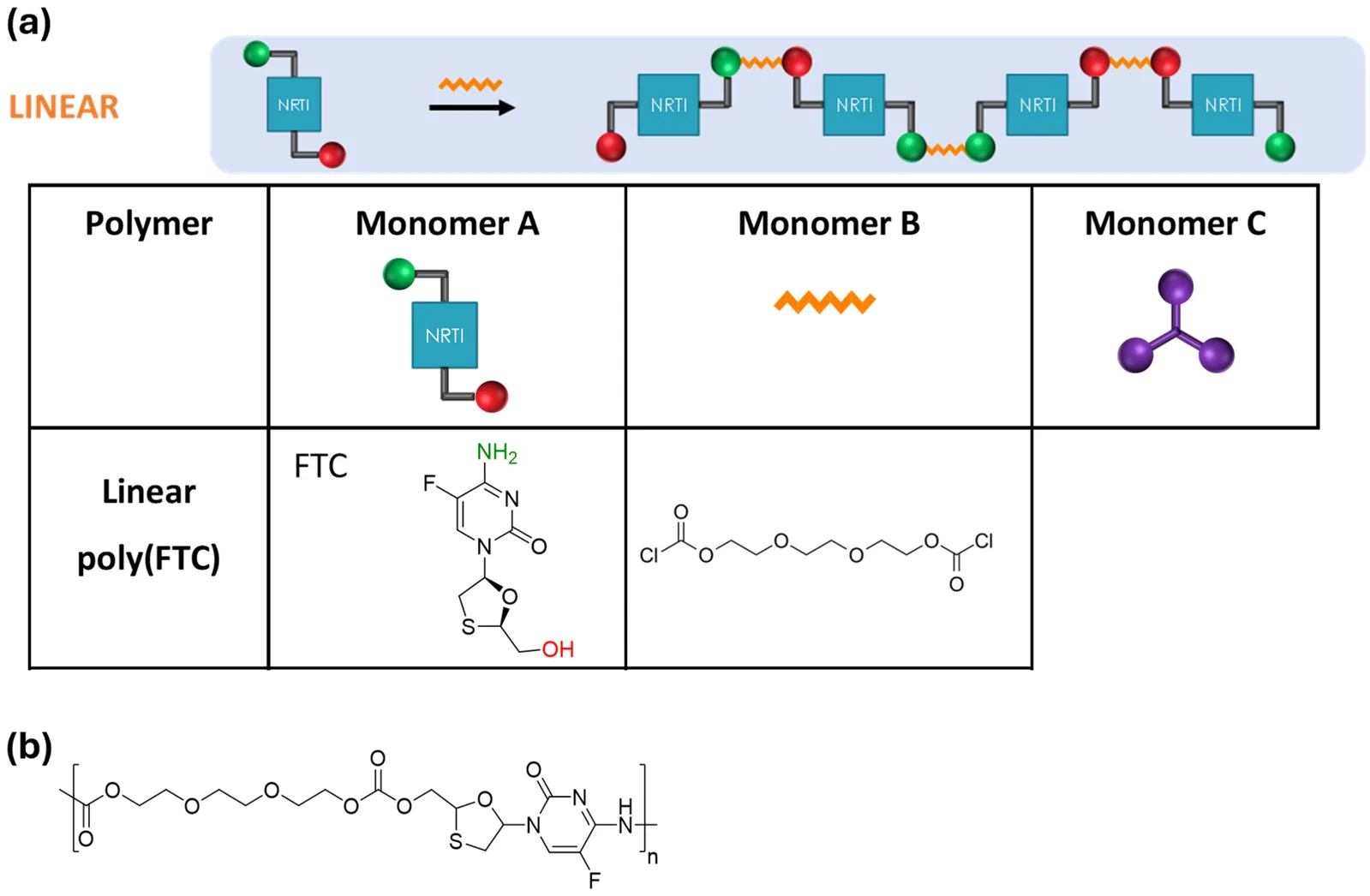
(**a**) POP materials strategy for polymer generation for FTC. On the FTC, the green end indicates the amine functional group, and the red end indicates the hydroxyl functional group. On the polymers formed from the linear method of polymer formation, the green end indicates the carbamate group formed, and the red end indicates the carbonyl group. (**b**) The linear poly(FTC) repeating unit is shown schematically; full synthesis, structural characterisation and degradation analyses have been reported previously [8].

The synthesis of Linear poly(FTC) materials was inspired by strategies for carbamate/carbonate prodrugs of FTC [15] and conducted as previously reported [8,16]. The orientation of the FTC and ordering of carbamate and carbonate linkages in the resulting polymers are statistical along the backbone. Linear poly(FTC) was prepared as a linear step-growth polymer (Figure 1). We did not perform any additional monomer-specific quality-control or structural analysis as part of the present immunocompatibility study.

Linear poly(FTC) was weighed and suspended at 20 mg/mL in DMSO. DMSO was chosen because it is commonly used as a vehicle for compounds insoluble in water for cellular assays and is generally well tolerated. FTC was weighed and suspended at 1 mg/mL in cell culture media. These were both freshly prepared and suspended at the start of each new assay. DMSO concentrations for the assayed linear poly(FTC) concentrations were as follows: cytotoxicity 1–0.002% (0.2–0.000391 mg/mL), pyrogen assessment 0.05/0.5% (10/100 μg/mL), and all other assays 0.2/0.4% (40/80 μg/mL).

### 2.3. Pyrogen Assessment of Linear Poly(FTC) Using the Monocyte Activation Test

The POP material in DMSO had a cloudy appearance, which would interfere with the Limulus Amoebocyte Lysate (LAL) chromogenic or turbidimetric assays and consequently, the monocyte activation test (MAT) was used to assess for pyrogens in the POP linear poly(FTC). The PyroMAT^®^ kit was carried out on linear poly(FTC) at final concentrations of 10 and 100 µg/mL, following the manufacturer’s protocol and already published methods [17].

An endotoxin standard curve was prepared using LAL reagent water. The United States Pharmacopoeia Reference Standard Endotoxin (RSE) stock solution (2000 EU/mL) was diluted to get a concentration of 0.8 EU, and seven endotoxin concentrations were prepared using 1:2 dilutions (0.8–0.0125 EU/mL) to generate the standard curve. To each of the two dilutions of linear poly(FTC), RSE was additionally spiked at a final concentration of 0.2 EU/mL. 50 μL of the blank standards and samples were added to the corresponding wells and assayed in quadruplicate.

Two vials of PyroMAT^®^ cells from cryogenic storage were thawed in a water bath at 37 °C, 1 mL of pre-warmed RPMI-1640 media was added to each vial and transferred into respective tubes with more media. The tubes were then spun at 210× *g*-force (g) for five minutes at 15–30 °C, the supernatants discarded and suspended in the remaining media and the two tubes combined. Warmed RPMI-1640 was then added to a total volume of 20 mL, and 200 μL of cells was added to every well on the plate, which was then incubated for 22 h at 37 °C, 5% CO_2_.

All components included in the IL-6 ELISA kit were warmed to 15–30 °C, and the manufacturer’s protocol was followed. A total of 100 µL of assay diluent was added to all the wells being used on the 96-well plate. A total of 100 µL of the cell supernatant from the set-up plate was then added to the corresponding wells on the ELISA plate; the plate was sealed and incubated at 15–30 °C for two hours. Wash buffer was made up by diluting the concentrate 1:25 with purified water. The plate was then aspirated and washed four times by adding 400 µL of wash buffer each time. Following final aspiration, 200 µL of IL-6 conjugate was added to each well; the plate was sealed and incubated for two hours at 15–30 °C. Following incubation, the wash step was repeated, and the substrate solution was prepared by mixing the contents of the colour reagent A and B bottles and inverting to mix. A total of 200 µL of substrate solution was then added to each well and incubated at 15–30 °C for 20 min, protected from light. A total of 50 µL of stop solution was added to each well and mixed by pipetting up and down. The optical density (OD) at 450 nm and a reference wavelength of 630 nm were read using the CLARIOstar plate reader.

### 2.4. Cytotoxicity Assessment of FTC, Linear Poly(FTC) Using the MTT and Lactate Dehydrogenase (LDH) Assays

The human peripheral blood acute monocytic leukaemia cell line, THP-1 cells were maintained in a routine cell culture medium of RPMI-1640 supplemented with 10% FBS.

MTT and LDH cytotoxicity experiments were undertaken as previously published [10,14]. A 1:2 dilution was performed across the plate, to create 9 or 10 final well concentrations in triplicate, ranging from 0.2 to 0.000391 mg/mL for the POP material linear poly(FTC), 250 to 0.977 μg/mL for FTC and 2 to 0.004% DMSO, which was also included as a vehicle control. Cells were plated at 5 × 10^4^ cells/well, and a cell-free treated control was set up in singlicate to ensure there was no material-assay interference. Each plate was set up in duplicate for each drug (half plate equals one experimental replicate with three technical replicates), and two plates were used for each drug for each cell line. Two untreated cell-containing columns and a media control column were also included. The plates were incubated for 24 h at 37 °C. Following incubation 5 µL of Lysis buffer from the kit was added to one of the untreated columns to create a cell death control and incubated for a further 15 min on a plate shaker set to 250 RPM at 37 °C. Plates were subsequently centrifuged at 860× *g* for five minutes, and the supernatants were transferred into 96-well flat-bottom plates for the LDH assay.

MTT powder (thiazolyl blue tetrazolium bromide dye; Sigma-Aldrich, USA) was prepared in PBS at 5 mg/mL and 50 mL was added to the cell pellet in each well and incubated for two hours at 37 °C, 5% CO_2_. Following incubation, the MTT plates were subsequently centrifuged at 1935× *g* for five minutes followed by aspiration. A total of 100 µL of DMSO was then added, mixed and the OD was measured at absorbances of 570 nm and 620 nm using the CLARIOstar plate reader.

All contents of the Cytotoxicity Detection Kit^PLUS^ (LDH) were allowed to warm up to 15–30 °C, and the manufacturer’s protocol was followed. The reaction mixture was prepared by adding 1 mL of sterile water to bottle 1; the contents were transferred into bottle 2 and vortexed. A total of 100 µL of freshly prepared reaction mixture was added to every well on the plate and incubated for 30 min at 15–30 °C in the dark. A total of 50 µL of stop solution was added to all wells; the plate was shaken for ten seconds, and OD was measured at absorbances of 493 nm and 600 nm using the CLARIOstar plate reader. GraphPad Prism software 10.2.3 was used to calculate effective concentration (EC)_20_ values for drugs showing complete cytotoxicity for each assay.

### 2.5. Leukocyte Proliferation Assessment of POP Material Linear Poly(FTC) and FTC

The Leukocyte proliferation assay was performed as described in [18], except that buffy coats were used instead of volunteer blood. Buffy coats were obtained from the National Health Service (NHS) blood and transport (BT) service. The blood was layered on top of Ficoll-Paque in a 1:2 ratio and then spun at 860× *g* for 30 min without break. The PBMC layer was then removed, washed with PBS and spun at 860× *g* for five minutes. The pellet was then suspended in 50 mL of RPMI-1640 with 10% FBS and placed in a T175 flask overnight. Each test polymer was diluted in media to 2× final concentration, and 100 µL was dispensed into the respective wells. Final concentrations of the test compounds were linear poly(FTC) 40/80 µg/mL (DMSO 0.4/0.2%) and FTC 1.8/18 µg/mL; these were set up in sextuplicate. FTC final concentrations reflected 1× and 10× recorded Cmax values on the HIV drug interactions website (FTC = 1.8 µg/mL) [19]. Linear poly(FTC) concentrations were chosen as they were not cytotoxic and had <0.5% DMSO. The concentrations were selected for comparative acute in vitro screening and do not represent validated estimates of in situ implant exposure. The conditions spiked with PHA-M at a final concentration of 5 μg/mL were also set up in sextuplicate. Controls were set up in duplicate at final concentrations of PHA-M 2.5, 5 and 10 μg/mL, and a DMSO control matching the highest linear poly(FTC) concentration (DMSO 0.4%). The cells were then counted and adjusted to a density of 1 × 10^6^ cells/mL. A total of 100 µL of cells or media was then plated in the respective wells and incubated at 37 °C, 5% CO_2,_ for 72 h. The plates were then centrifuged at 700× *g* for five minutes, and the supernatant was aspirated. A total of 50 µL of MTT (5 mg/mL in PBS) and 200 µL of fresh media was added to each well and incubated for four hours at 37 °C, 5% CO_2_. Following incubation, the plates were centrifuged at 1935× *g* for five minutes and the supernatants aspirated. A total of 200 µL of DMSO was added to all wells, mixed and the OD was read at 570 nm and 620 nm. Statistical analysis was performed using GraphPad Prism software 10.2.3. Statistical comparisons were performed on log10-transformed values using ordinary one-way ANOVA with Dunnett’s multiple comparisons test (compare treatment conditions within donors from untreated) and repeated-measures ANOVA with Dunnett’s multiple comparisons test (compare treatment conditions from all donors from untreated). A *p* value < 0.05 was considered statistically significant.

### 2.6. Assessment of POP Material Linear Poly(FTC) on Complement Activation

The complement assay was adapted from [9,20], with the major change being the use of hirudin as the anticoagulant of choice for these assays due to its ability to uncover weaker complement activators [21]. Blood was collected in hirudin-coated collection tubes from healthy volunteers at the University of Liverpool through venipuncture by trained staff, with all protocols and procedures approved by the University Research Ethics Committee (REC) approval number 11499, termed the Pharmacology Biobank (PHARM B). Within 30 min of being drawn, the blood was centrifuged at 2500× *g* for ten minutes. Samples were set up in duplicate for each donor with 100 μL of PBS, and 100 μL of test compound was diluted in PBS and 100 μL of fresh plasma. Final concentrations of the test compounds were linear poly(FTC) 40/80 µg/mL (DMSO 0.4%), and FTC 1.8/18 µg/mL. Alongside this vehicle control (0.4% DMSO), an untreated control (PBS alone) and two positive controls were set up: Doxil at 200/16.42 µg/mL (total lipid content/Doxorubicin content) and CVF at 1035 units/mL. All the Eppendorf tubes were vortexed and then incubated statically at 37 °C for 30 min. Following the incubation period, the samples were immediately analysed in duplicate using the MicroVue™ Complement iC3b EIA following the manufacturer’s protocol. Samples were diluted at either 1:40 or 1:20 in iC3b specimen diluent for the positive controls (CVF and Doxil) and all other samples, respectively, and assayed. A total of 100 μL of each of the blank, standards, controls, or samples was added to the corresponding wells of the 96-well plate in duplicate on the iC3b ELISA plates. OD was read at 405 nm, and the data was analysed by removing the blank and plotting the Concentration (*x*-axis) against absorbance (*y*-axis) and the test sample concentrations interpolated from the curve created using GraphPad Prism software 10.2.3. Statistical analysis was performed using GraphPad Prism software 10.2.3. Statistical comparisons were performed on log10-transformed values using ordinary one-way ANOVA with Dunnett’s multiple comparisons test (compare treatment conditions within donors to untreated) and repeated-measures ANOVA with Dunnett’s multiple comparisons test (compare experimental control conditions to all donors to untreated or treatment conditions from all donors from vehicle control). A *p* value < 0.05 was considered statistically significant.

Factors H, Bb, C4d and sC5b-9 were also measured from these samples using the Quidel EIAs, and the manufacturer’s protocol was followed. The following dilutions were used: Factor H, all samples were diluted 1:5000; Bb, all samples were diluted 1:75; C4d, all samples were diluted 1:30; sC5b-9, all samples diluted 1:100 except positive controls (CVF/Doxil), which were diluted 1:400.

### 2.7. Assessment of POP Materials in Whole Blood Using Control Ligand Panel

The whole blood assay was adapted from the PBMC cytokine secretion assays used previously by the group [9,22]. Blood was collected fresh in tubes containing the anticoagulant Li-heparin from health volunteers using the PharmB ethics. Within 30 min of being drawn, the blood was diluted 1:4 with complete culture media (RPMI supplemented with 10% FBS); 800 µL of diluted blood was then seeded into 24-well plates and 200 µL of each diluted test compound was added. The following final concentration of the agonist compounds set up were: LPS 20 ng/mL, the principal component of Gram-negative bacteria, which acts on and activates TLR-4, PHA-M 10 µg/mL, a potent mitogen that crosslinks the human T lymphocyte antigen receptor (Ti), media (Untreated), ODN 2006 38.5 ng/mL, contains CpG motifs that act on and activate TLR-9, 2′3′-cGAMP 10 µg/mL, a STING agonist, Poly(dG:dC)/LyoVec™ 5 µg/mL, HSV-60/LyoVec™ 5 µg/mL, both act on Cytosolic DNA sensors (CDS). The final concentrations of the test compounds set up were linear poly(FTC) 40/80 μg/mL (DMSO 0.4%) and FTC 1.8 μg/mL. The samples were incubated for 24 h at 37 °C, 5% CO_2_. Samples were then centrifuged at 860× *g* for five minutes, and 100 µL aliquots of the supernatants were frozen at −80 °C until analysis.

The supernatants were thawed and cytokine analysis was carried out following the Human Magnetic Luminex Assay protocol. Kit reagents were brought up to room temperature. Each standard provided in the kit was reconstituted with Calibrator Diluent RD6-52 using the volumes specified on the certificate of analysis and allowed to stand for 15 min with gentle agitation. A total of 100 µL of each were then combined with Calibrator Diluent RD6-52 to a final volume of 1 mL to create standard 1. A 3-fold dilution series was performed using calibrator Diluent RD6-52 to create standards 2–6. Samples were centrifuged at 860× *g* for five minutes, and 50 µL of sample or standards were plated in their respective wells and analysed. A total of 500 µL of human magnetic microparticle cocktail was added to 5 mL of Diluent RD2-1 and 50 µL added to every well and incubated at 15–30 °C on a plate shaker set at 800 RPM for two hours. Wash buffer concentrate was diluted 1:25 with distilled water and the plate was washed three times with addition of 100 µL of wash buffer. A total of 500 µL Biotin-antibody cocktail was diluted with 5 mL of Diluent RD2-1 and 50 µL added to each well and incubated at 15–30 °C on the plate shaker for one hour. The plate wash was repeated, 220 µL of streptavidin-PE concentrate was added to 5.35 mL of Wash buffer, 50 µL added to each well and incubated at 15–30 °C on a plate shaker for 30 min. The plate wash was repeated and 100 µL of wash buffer was added to each well and incubated for two minutes on a plate shaker. The plate was analysed using the Bio-Plex 200 Luminex (Bio-Rad Laboratories, Hemel Hempstead, UK) following calibration, setting the sample volume at 50 µL, bead type as Bio-Plex MagPlex Beads, set double discriminator gates at 8000 and 23,000, reporter gain settings set to low RP1 target value for CAL2 setting, 50 counts/region and collect median fluorescence intensity (MFI). The minimum and maximum limits of quantification for these cytokines are displayed in Table 1. Statistical analysis was performed using GraphPad Prism software 10.2.3. Statistical significance was evaluated using a Friedman test with Dunn’s multiple comparisons test (compare treatment conditions from all donors from untreated).

The average concentrations calculated for each cytokine were plotted, values that were above the limit of quantification were plotted at the limit of detection and values below the limit of detection were plotted as 0. Samples that had one replicate within the range were plotted at the value that was in range alone. A dashed line has been used to indicate where these limits of detection are on the graphs that contained samples that were either above or below the limit of detection. GraphPad Prism software 10.2.3 was used to display the data.

## 3. Results

### 3.1. Pyrogen Assessment of POP Materials, Measured by MAT Assay, Shows No Apparent Pyrogen Contamination

**Figure 2:**
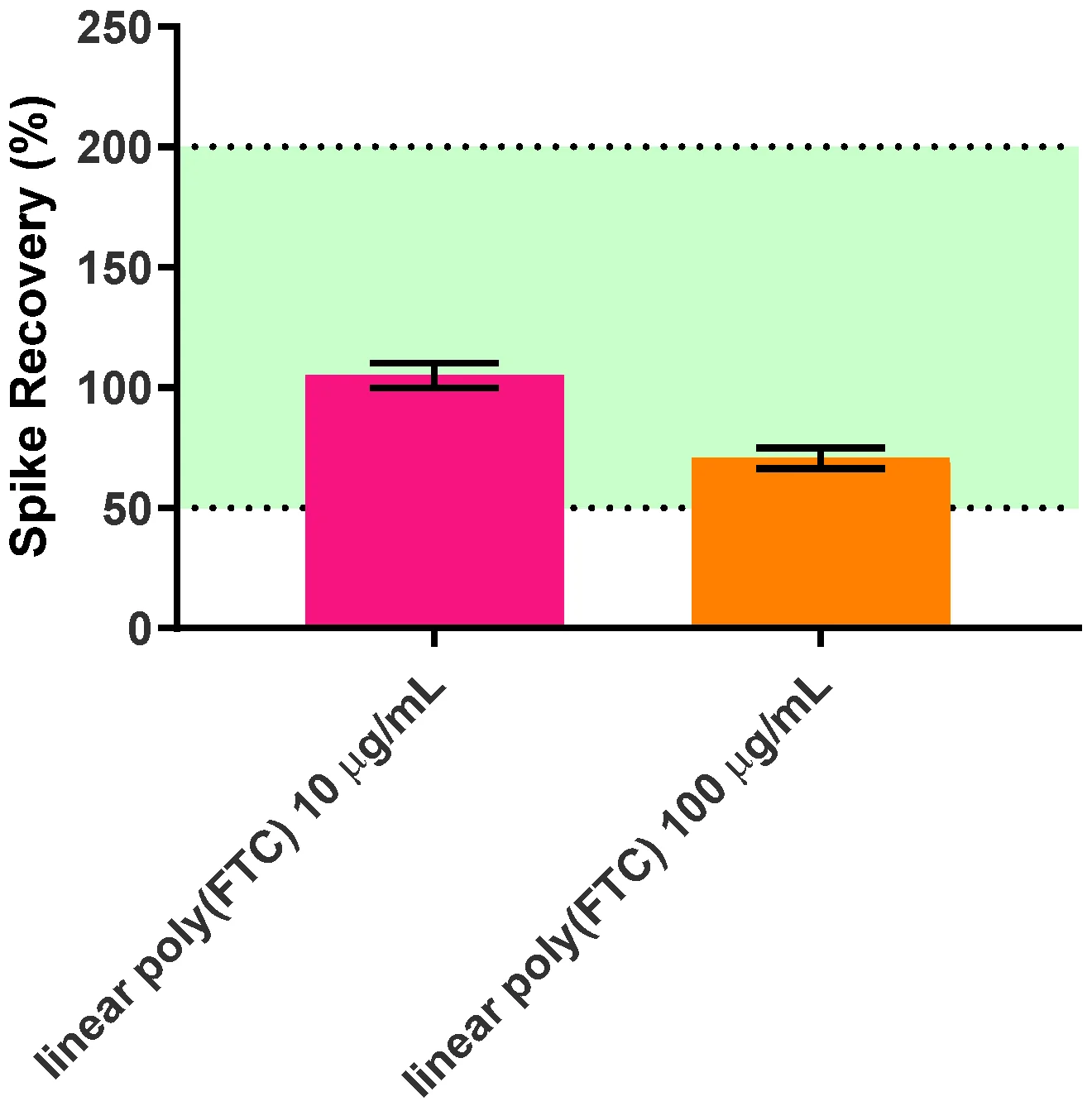
MAT recovery of RSE from samples of linear poly(FTC) at 10 and 100 µg/mL, spiked with RSE at a final concentration of 0.2 EU/mL. All unspiked samples had undetectable IL-6 levels. Data displayed as *n* = 4, mean (±standard deviation). Spike recovery: green area indicates 50–200% recovery following LPS spike.

The MAT assay was used to detect whether the materials tested contained pyrogens. No detectable pyrogenic response was observed in unspiked linear poly(FTC) samples under the assay conditions. Both concentrations of the linear poly(FTC)-spiked samples had between 50 and 200% endotoxin spike recoveries; 50–200% is the acceptable range of recovery for the assay (Figure 2). The average % recovery for linear poly(FTC) at 10 µg/mL was 105%, and at 100 µg/mL, was 70.67%. This data suggests that the level of pyrogen contamination in the sample is acceptable.

### 3.2. Cytotoxicity Assessment of FTC and Linear Poly(FTC) in THP-1 Cells Shows No Overt Cytotoxicity Under the Conditions Tested

Cytotoxicity assessment was carried out on the antiretroviral FTC and POP linear poly(FTC), alongside DMSO, the vehicle used for linear poly(FTC), to find appropriate concentrations to test in subsequent assays.

**Figure 3:**
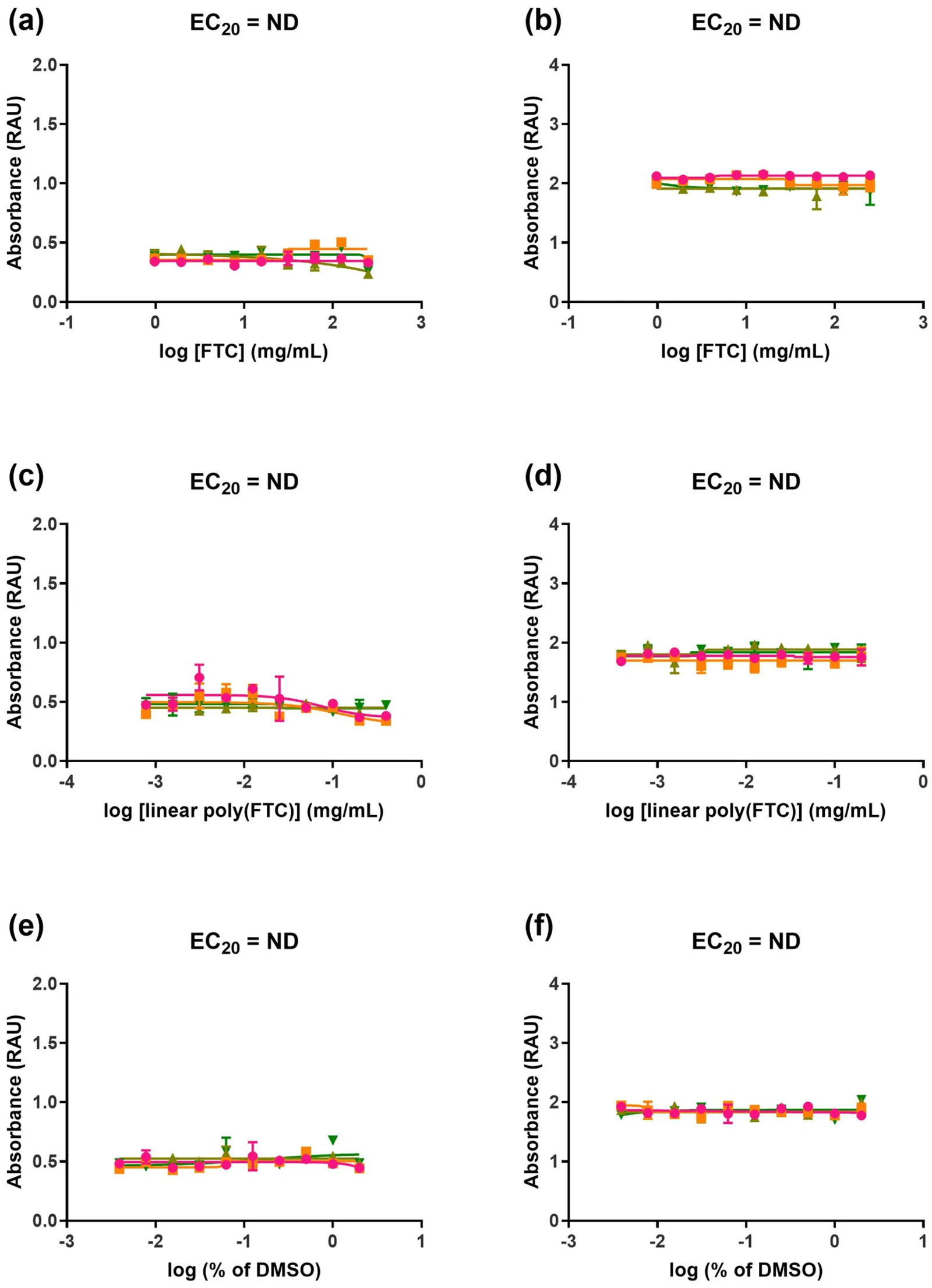
Cytotoxicity assessment of FTC in THP-1 cells following 24 h exposure: (**a**) FTC MTT, (**b**) FTC LDH. (**c**) Linear poly(FTC) MTT, (**d**) linear poly(FTC) LDH. (**e**) DMSO MTT, (**f**) DMSO LDH. Data displayed *n* = 3 technical replicates mean (±Standard Deviation) and *n* = 4 experimental replicates, each in a different colour. Not determined (ND) is used to describe EC_20_ values that could not be computed due to incomplete cytotoxicity curves.

FTC caused no overt toxicity at the concentrations tested in the MTT or LDH assay over 24 h (Figure 3a,b). Linear poly(FTC) also caused no overt toxicity in either of the cytotoxicity assays (Figure 3c,d). DMSO at the percentages tested did not cause any overt toxicity in either the MTT or LDH assay over 24 h, and therefore, EC_20_ values could not be calculated (Figure 3e,f). Neither FTC nor linear poly(FTC) caused overt loss of metabolic activity or membrane integrity over 24 h, so EC_20_ values could not be calculated within the tested range; 40/80 µg/mL was taken forward for linear poly(FTC), and 1.8/18 μg/mL was taken forward for FTC where possible.

### 3.3. Leukocyte Proliferation Assessment of POP Material Linear Poly(FTC) Shows Interindividual Variability in Responses

**Figure 4:**
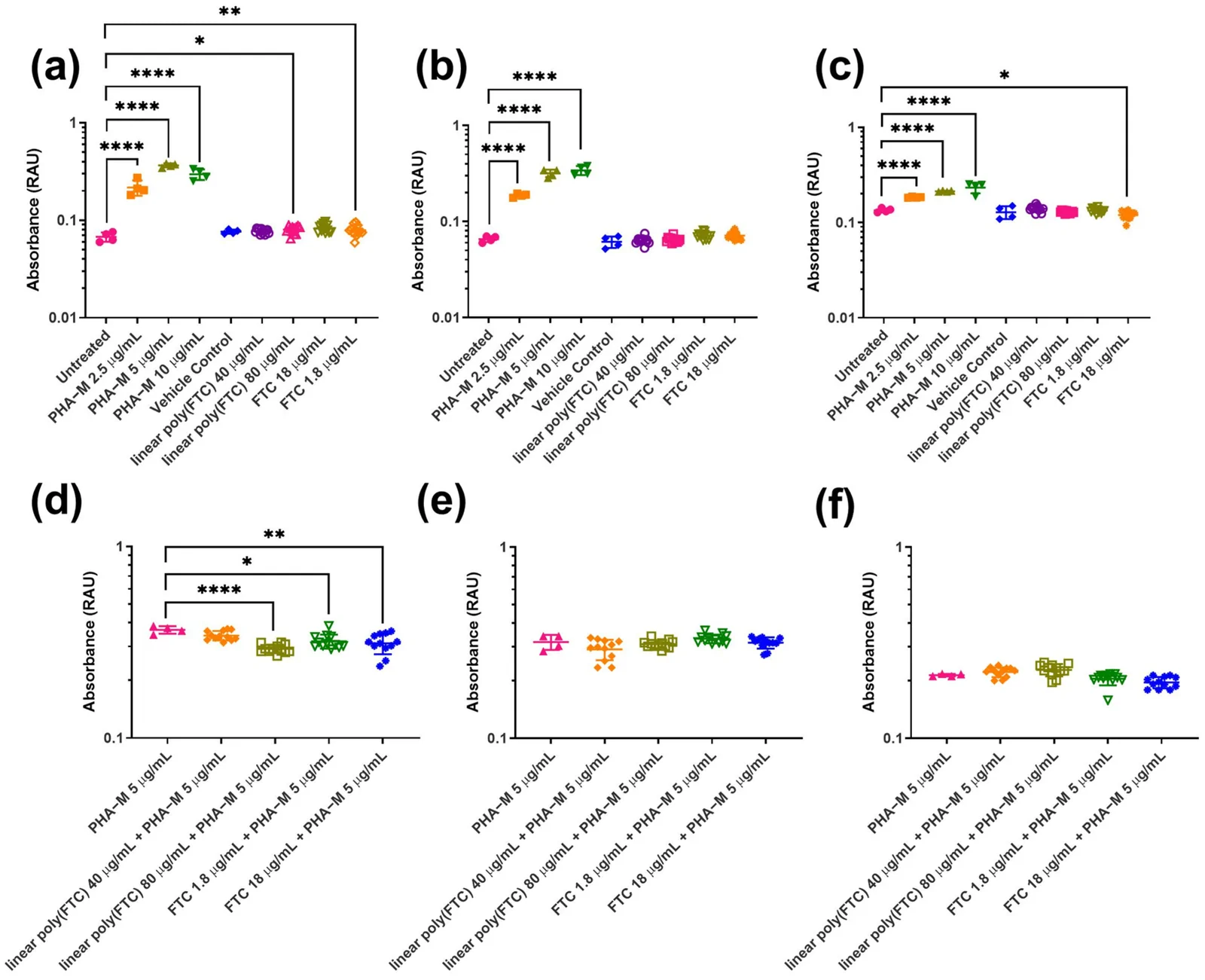
Leukocyte proliferation, as measured by MTT, in PBMCs exposed to linear poly(FTC) and FTC. (**a**,**d**) Donor 1, (**b**,**e**) Donor 2, (**c**,**f**) Donor 3. Data displayed as *n* = 4 for untreated, each PHA-M concentration, vehicle control, *n* = 12 for each of the linear poly(FTC)/FTC alone and their spiked samples, mean (±standard deviation). *p* < 0.0001 = ****, *p* < 0.01 = ** and *p* < 0.05 = *.

**Figure A1:**
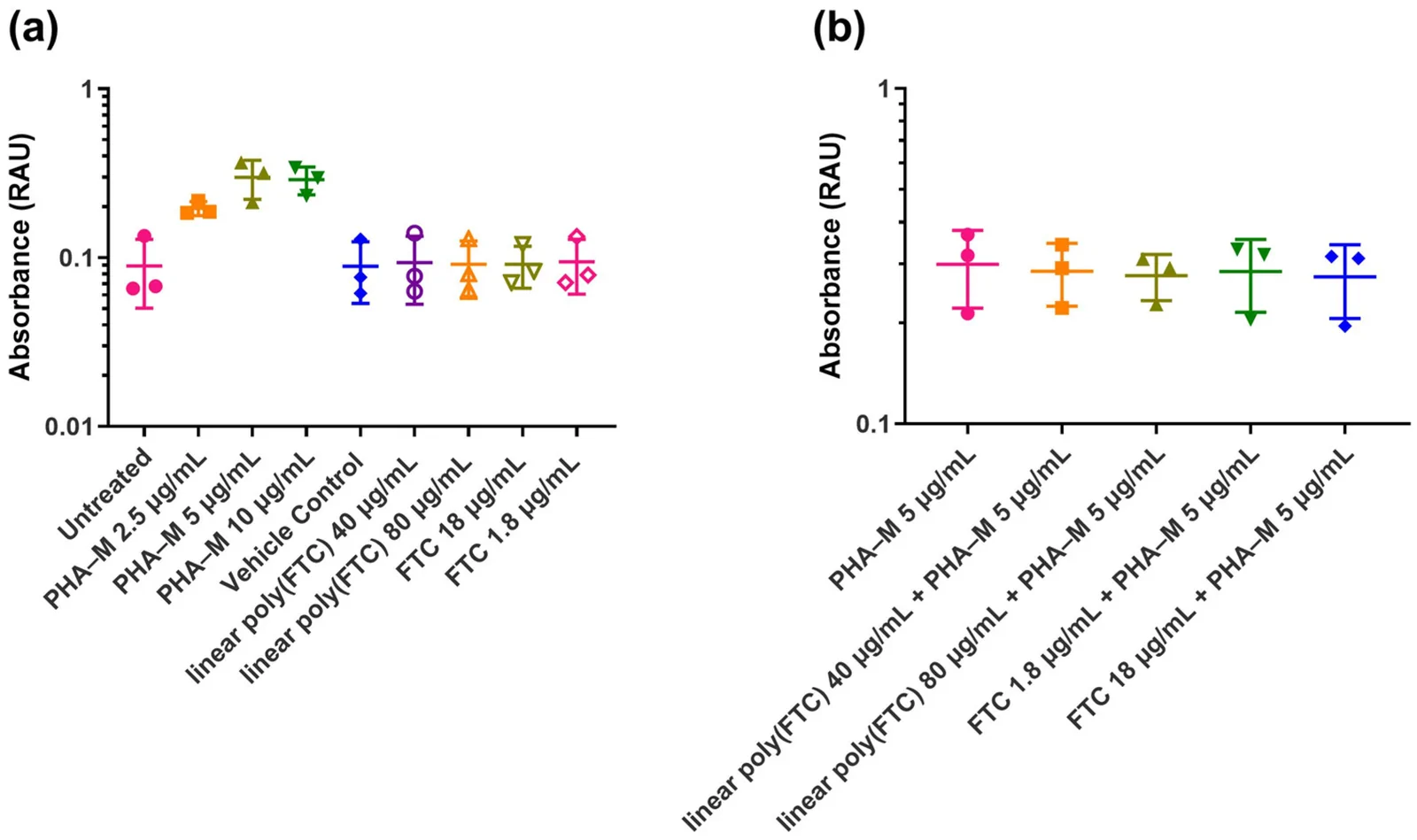
Leukocyte proliferation, as measured by MTT in PBMCs exposed to linear poly(FTC) and FTC. Data displayed as *n* = 3 donors, mean (±standard deviation). (**a**) Shows treated samples, (**b**) shows treated samples spiked with PHA-M at a final conc.

Leukocyte proliferation was assessed to explore the ability of FTC or linear poly(FTC) to screen for potential immune activation. All three concentrations of PHA-M showed a significantly higher absorbance compared to the untreated control in each donor and, therefore, proliferation when compared to the untreated cells (Figure 4a–c) (*p* < 0.0001). In donor 1, significantly higher absorbance was seen for linear poly(FTC) 80 μg/mL and FTC 1.8 µg/mL (*p* < 0.05 and *p* < 0.01) when compared to the untreated (Figure 4a). Donor 3 also showed significantly higher absorbance for FTC 18 μg/mL (*p* < 0.05) (Figure 4c). None of the controls or tested conditions alone (linear poly(FTC), FTC or the vehicle control) caused any significant proliferation when combined donor data were compared to the untreated (Figure A1).

No significant alterations between the PHA-M 5 µg/mL control and the PHA-spiked (5 µg/mL) samples of FTC and linear poly(FTC) were seen when all donors were combined (Figure A1). In individual donors, leukocyte proliferation data was unaffected by either PHA-spiked samples of FTC or linear poly(FTC) at the concentrations tested in donors 2 and 3; however, in donor 1, there were some significant alterations in the PHA-spiked samples of FTC and linear poly(FTC) 80 μg/mL (Figure 4d).

### 3.4. Linear Poly(FTC) Induces Donor-Dependent Levels of iC3b Generation in Human Plasma

Complement assessment using the surrogate iC3b was used to determine the ability of FTC and linear poly(FTC) to interact with the complement system. The complement components Bb, C4d, Factor H and sC5b-9 were subsequently measured in an attempt to determine the route of complement activation.

**Figure 5:**
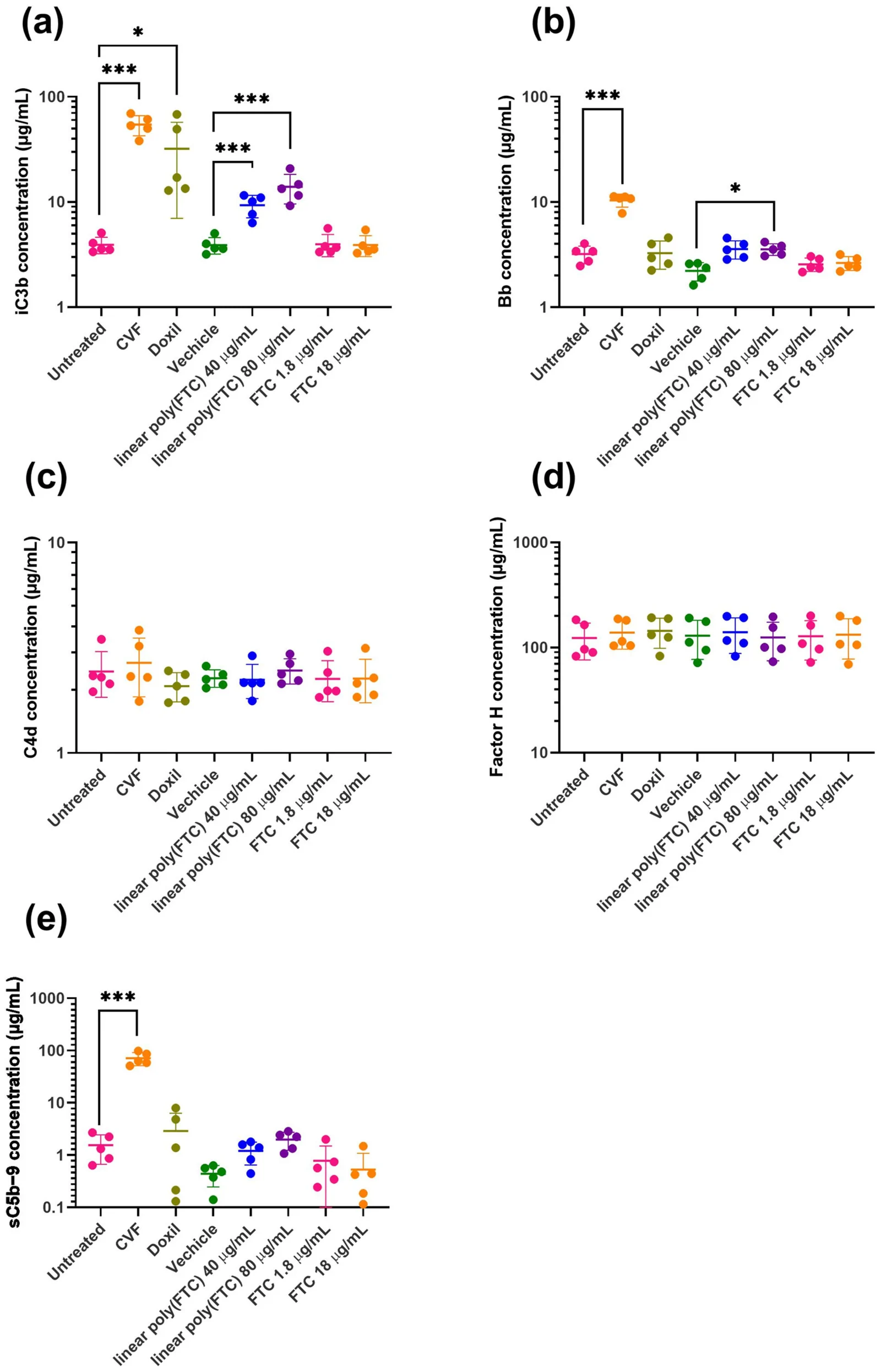
Complement activation, as measured by complement component EIAs in plasma samples exposed to linear poly(FTC) and FTC. (**a**) iC3b, (**b**) Bb, (**c**) C4d, (**d**) Factor H, (**e**) sC5b-9. Data displayed as average of two technical replicates for *n* = 5 donors, mean (±standard deviation). *p* < 0.001 = *** and *p* < 0.05 = *.

**Figure A2:**
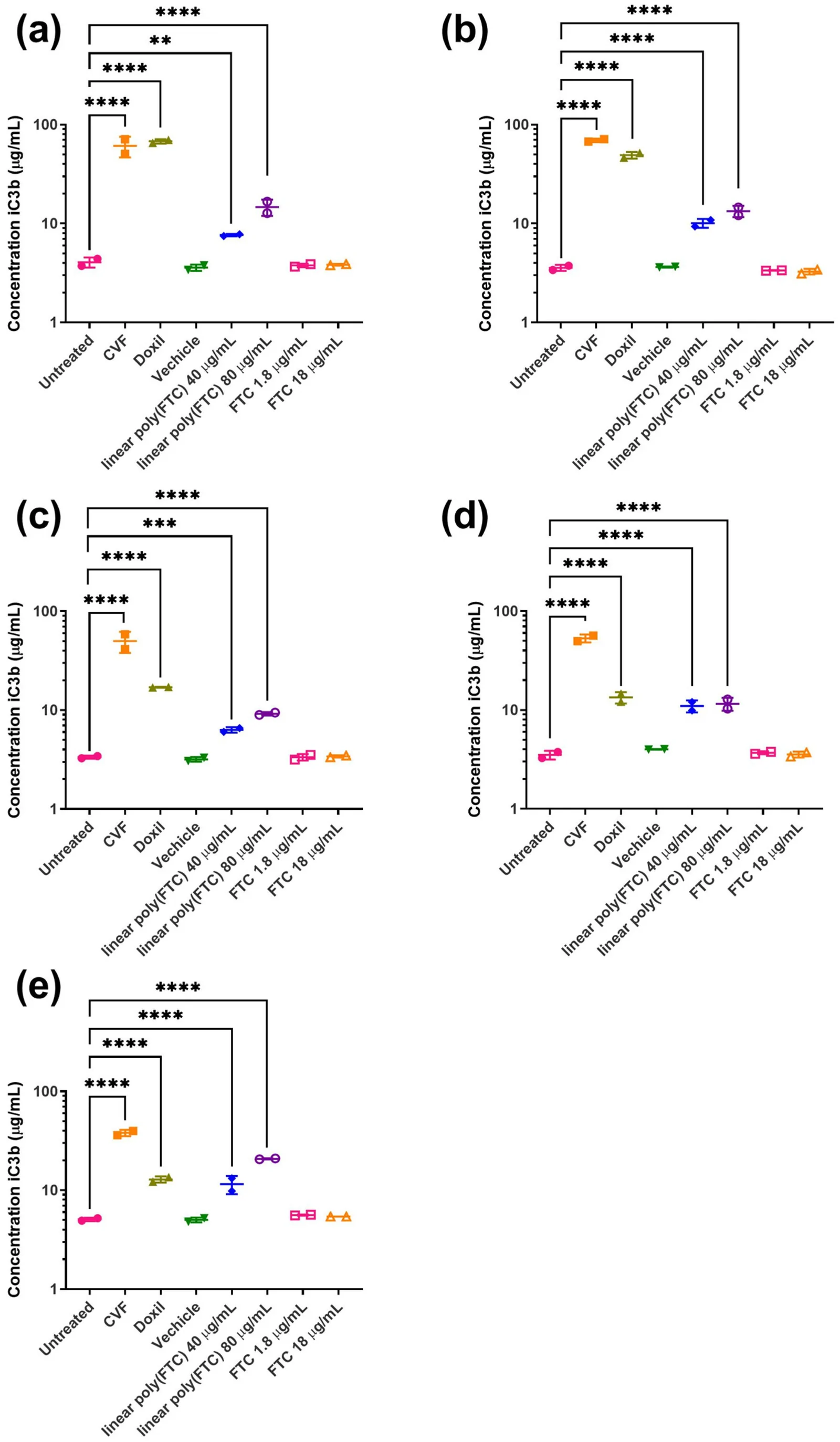
Complement activation, as measured by iC3b EIA in plasma samples exposed to linear poly(FTC) and FTC. (**a**) Donor 1, (**b**) Donor 2, (**c**) Donor 3, (**d**), Donor 4, (**e**) Donor 5. Data displayed as average of *n* = 2 ± standard deviation. *p* < 0.0001 = ****, *p* < 0.001 = *** and *p* < 0.01 = **.

When the average concentration of iC3b for each of the five donors is combined, CVF and Doxil show significantly higher iC3b concentrations when compared to the untreated (*p* < 0.001 and *p* < 0.05, respectively) (Figure 5a). When compared individually, CVF caused a higher iC3b concentration in all five donors, with between 649% and 1842% higher iC3b concentrations when compared to the untreated (Figure A2). Doxil also caused higher iC3b concentrations when compared to the untreated, with between 154% and 1567% higher iC3b concentrations when compared to the untreated (Figure A2).

When the average concentration of iC3b for each of the five donors is combined, linear poly(FTC) 40 μg/mL and 80 μg/mL show significantly higher concentration-associated iC3b concentrations when compared to the untreated (*p* < 0.01 and *p* < 0.001, respectively) (Figure 5a). When comparing the linear poly(FTC) 80 µg/mL treatments to the vehicle (0.4% DMSO), the statistical significance remained the same as compared to the untreated with 80 µg/mL; however, linear poly(FTC) 40 µg/mL showed a significantly higher iC3b concentration when compared to the vehicle (0.4% DMSO, not matched as 40 µg/mL contained 0.2% DMSO) (*p* < 0.001). Individual donor iC3b data is displayed in Figure A2. When compared individually, linear poly(FTC) caused a higher iC3b concentration in all five donors with varying *p* values; 40 μg/mL ranged from 89% to 213%, higher than the untreated, and 80 μg/mL ranged from 175% to 309% (Figure A2). When comparing the linear poly(FTC) 80 µg/mL treatment to the vehicle (0.4% DMSO), the statistical significance remained the same as when comparing to the untreated.

When the average concentration of Bb for each of the five donors is combined, CVF showed significantly higher Bb concentrations when compared to the untreated (*p* < 0.001) (Figure 5b). When the average concentration of C4d for each of the five donors is combined, no treatments show significantly higher C4d concentrations when compared to the untreated (Figure 5c). When the average concentration of Factor H for each of the five donors is combined, no treatments show significantly higher Factor H concentrations when compared to the untreated (Figure 5d). When the average concentration of sC5b-9 for each of the five donors is combined, CVF showed significantly higher sC5b-9 concentrations when compared to the untreated (*p* < 0.001) (Figure 5e). When comparing the linear poly(FTC) treatments to the vehicle (0.4% DMSO), the statistical significance remained the same, with the following exception: linear poly(FTC) 80 µg/mL showed a significantly higher Bb concentration when compared to the vehicle (0.4% DMSO).

Doxil showed high interindividual variation in complement activation and linear poly(FTC) caused a donor-dependent concentration-associated increase in complement, as measured by iC3b levels.

### 3.5. Assessment of Whole Blood Responses to Linear Poly(FTC)

**Figure 6:**
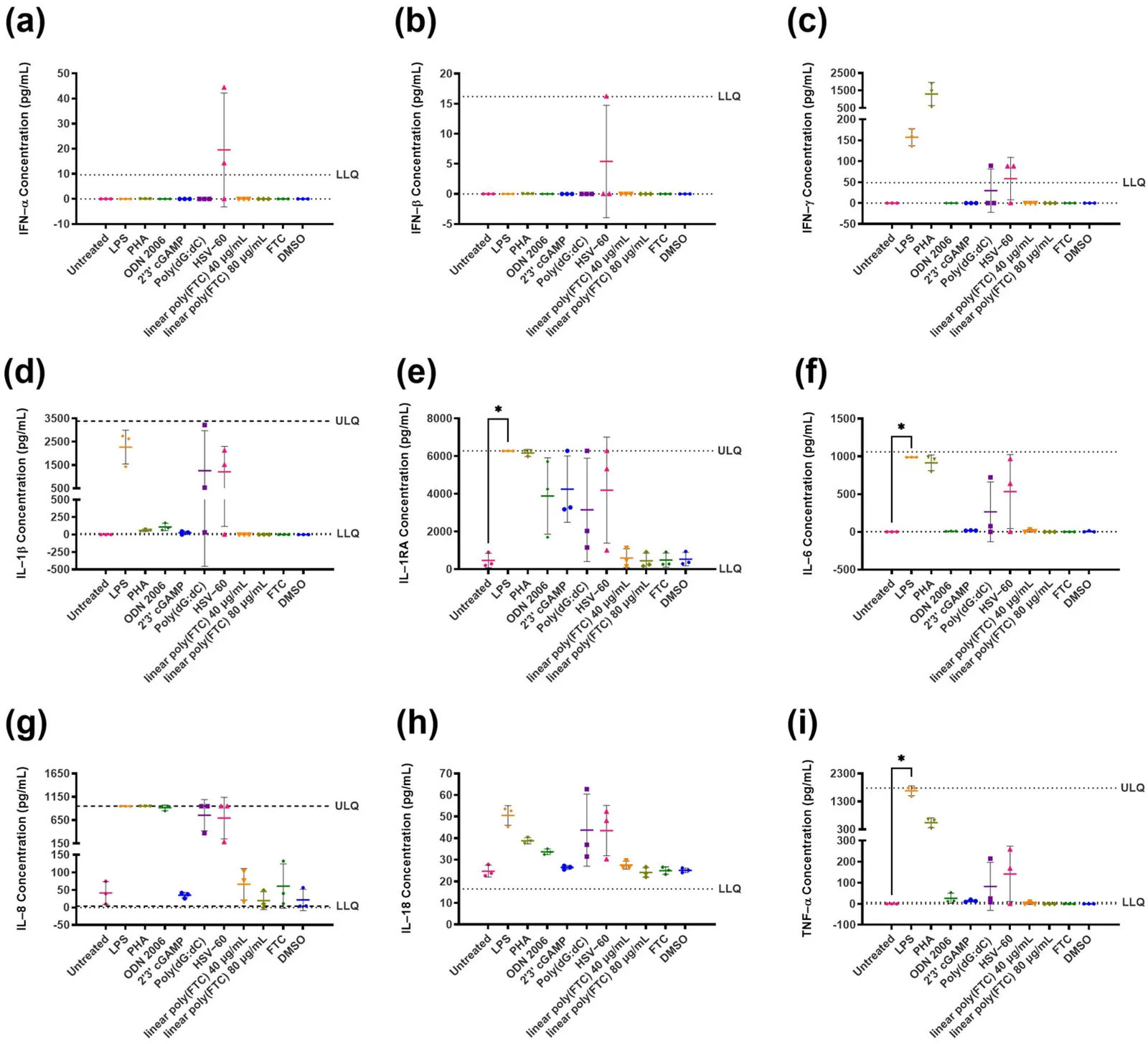
Whole-blood cytokine concentrations of supernatants treated with control agonists, POP material linear poly(FTC) and FTC. (**a**) IFN-α, (**b**) IFN-β, (**c**) IFN-γ (**d**) IL-1β, (**e**) IL-1RA, (**f**) IL-6, (**g**) IL-8, (**h**) IL-18 and (**i**) TNF-α. Data displayed *n* = 3 donors, mean (±standard deviation). Dashed lines ULQ indicate the upper limit of the standard curve and dashed lines LLQ indicate the lower limit of the standard curve. *p* < 0.05 = *.

A small panel of PRR agonists were used to ensure that whole blood was viable and responsive (Figure 6). Neither FTC nor linear poly(FTC) treated blood samples led to significantly higher secretion of any of the nine cytokines measured (Figure 6).

## 4. Discussion

This study contains an early acute immunocompatibility assessment of a POP material—linear poly(FTC). Determining the immunocompatibility profile of this material is critical for its further development and eventual clinical translation.

Because linear poly(FTC) was cloudy and would interfere with the LAL assay, we employed the MAT to detect endotoxin and pyrogen contamination. The MAT assay did not reveal significant IL-6 production and therefore the MAT did not detect pyrogenic activity under the assay conditions. Although samples spiked with endotoxin at 100 µg/mL showed reduced percentage recovery—possibly due to cell death—the recovery values remained within the acceptable 50–200% range. Moreover, whole blood assays did not exhibit significant increases in IL-8 or TNF-α, supporting a lack of broad inflammatory activation. These findings allowed us to advance the material into subsequent immunocompatibility evaluations.

The leukocyte proliferation assay, which measures the responses of monocytes and T and B lymphocytes, is a key indicator of immunological homeostasis. Neither linear poly(FTC) nor FTC, combined with PHA-M, induced significant leukocyte proliferation when comparing all three donors together; however, there were small but significantly higher results for FTC in donors one and three, as well as for linear poly(FTC) at 80 µg/mL in donor one. Notably, one donor showed a reduction in absorbance—suggesting decreased leukocyte numbers—when exposed to 80 µg/mL linear poly(FTC) and to 1.8 or 18 µg/mL FTC combined with PHA. This observation may indicate an inhibitory effect on PHA-induced activation; however, further investigation with a larger cohort is required because it was significant in only one of the three donors tested. If confirmed in vivo, such inhibition could potentially impair the host’s infection response.

Analysis of iC3b levels suggests that linear poly(FTC) weakly activates the complement system at the concentrations tested [20,21]. Hirudin was used as the anticoagulant in this assay to avoid ion chelation, thereby facilitating a more sensitive detection of complement responses and ensuring the complement pathways remain intact [21]. Higher Bb levels were observed in the linear poly(FTC) 80 µg/mL samples when compared to the vehicle-matched sample (0.4% DMSO), but not the untreated samples, suggesting potential for alternative pathway activation and warranting follow-up to confirm. The isolated iC3b and Bb increase is associated with linear poly(FTC) exposure without group-level C4d or sC5b-9, unlike expected results from CVF/Doxil, suggesting low-level C3 activation/processing and potential alternative pathway activation; however, we cannot identify the clinical relevance from this dataset and require follow up to confirm the pathway of activation.

The synthesis and degradation behaviour of linear poly(FTC) have been previously described. However, we did not characterise the chemical species present during the current immunocompatibility exposures. These experiments therefore cannot attribute the complement findings to specific degradation fragments, functional groups or antibody interactions. Future studies should combine chemical characterisation of released species with immunocompatibility assessment of defined degradation products, formed implants and implant extracts. The present experiments used DMSO-dispersed polymer and do not reproduce the surface properties, local exposure conditions or prolonged residence of a formed subcutaneous implant.

The selective increase in iC3b and Bb is consistent with C3 activation and subsequent C3b processing. However, iC3b generation depends on Factor I-mediated regulation of C3b, and DMSO has been reported to affect complement activity. Therefore, polymer-associated iC3b responses should be interpreted against matched DMSO vehicle controls, and further studies measuring C3a/C3a-desArg and additional pathway markers would be required to distinguish increased C3 activation from altered C3b processing, as well as intact polymer rods.

This study, however, does have some limitations: (1) it only contains an acute assessment of the Linear poly(FTC) POP and therefore does not explore the potential for long-term impacts on the immune system; (2) there are only three donors used for each assay, as opposed to the complement, which uses five donors, and therefore they all havea low probability of reflecting the responses of the whole population; (3) assessments only looked at Linear poly(FTC) using the vehicle DMSO and not a solid rod implant; (4) there is no assessment of the degradation products and therefore may not represent what is seen in vivo fully. The complement results did not indicate a pathway of activation, and therefore, the impact in humans is still unresolved.

## 5. Conclusions

Linear poly(FTC) did not induce detectable pyrogenic activity, overt cytotoxicity in the THP-1 cell line, consistent leukocyte proliferative activation, or broad whole-blood cytokine induction under the conditions tested. The complement assessment identified a selective increase in iC3b and Bb elevation at 80 g/mL compared to the vehicle (0.4% DMSO) without corresponding increases in C4d, Factor H or sC5b-9.

These findings are preliminary; due to the observed inter-donor variability, a larger donor cohort is required to draw definitive conclusions. The absence of elevated cytokine levels in response to FTC and linear poly(FTC) is promising. The precise degradation kinetics of these POP materials remain unpredictable at this early stage of development, necessitating further studies to assess the immunological impacts of the degradation products. Based on these results, further immunocompatibility studies—particularly those investigating long-term exposures and more complex immunological interactions—are justified.

## Figures and Tables

**Table 1:** Demonstrates the minimum and maximum limits of quantification for each panel of analytes in the whole blood assay assessment.

Analyte	Minimum (pg/mL)	Maximum (pg/mL)
IFN-α	9.63	2340
IFN-β	16.20	3390
IFN-γ	48.601	11,810
IL-1β/IL-1F2	13.951	3990
IL-6	4.074	990
IL-8/CXCL-8	3.909	950
IL-18/IL-1F4	16.543	4020
TNF-α	7.325	1780
IL-1 Receptor Antagonist (RA)	25.844	6280

## Data Availability

Supporting data is available from authors upon reasonable request.

## Abbreviations

The following abbreviations are used in this manuscript:

POP	Polymer of Prodrug
NRTI	Nucleoside Reverse Transcriptase Inhibitor
FTC	Emtricitabine
LATs	Long-acting therapeutics
TAF	Tenofovir Alafenamide
GMP	Good manufacturing practice
MAC	Membrane attack complex
PHA-M	Phytohaemagglutinin-M
MTT	Thiazolyl Blue Tetrazolium Bromide
DMSO	Dimethyl sulfoxide
RPMI	Roswell Park Memorial Institute
LDH	Lactate dehydrogenase
EIAs	Enzyme Immunoassays
CVF	Cobra venom factor
ODN	Oligodeoxynucleotide
HSV	Herpes Simplex virus
IFN	Interferon
RA	Receptor Antagonist
TNF	Tumour Necrosis Factor
FBS	Foetal Bovine Serum
LAL	Limulus Amoebocyte Lysate
MAT	Monocyte activation test
RSE	Reference Standard Endotoxin
OD	Optical density
EC	Effective concentration
NHS	National Health Service
BT	Blood and transport
REC	Research Ethics Committee
PHARM B	Pharmacology Biobank
Ti	human T lymphocyte antigen receptor

## References

[1] Swindells S., Andrade-Villanueva J.-F., Richmond G.J., Rizzardini G., Baumgarten A., Masiá M., Latiff G., Pokrovsky V., Bredeek F., Smith G. (2020). Long-Acting Cabotegravir and Rilpivirine for Maintenance of HIV-1 Suppression. N. Engl. J. Med..

[2] Pappa S., Emsley R. (2024). The evolution of long-acting antipsychotic treatments. Ther. Adv. Psychopharmacol..

[3] Darville N., van Heerden M., Mariën D., De Meulder M., Rossenu S., Vermeulen A., Vynckier A., De Jonghe S., Sterkens P., Annaert P. (2016). The effect of macrophage and angiogenesis inhibition on the drug release and absorption from an intramuscular sustained-release paliperidone palmitate suspension. J. Control. Release.

[4] Nakamizo S., Kabashima K. (2025). Cutaneous granulomas: Mechanisms, cellular interactions and therapeutic insights. Br. J. Dermatol..

[5] Sheikh Z., Brooks P.J., Barzilay O., Fine N., Glogauer M. (2015). Macrophages, Foreign Body Giant Cells and Their Response to Implantable Biomaterials. Materials.

[6] Zolezzi M., Abouelhassan R., Eltorki Y., Haddad P.M., Noorizadeh M. (2021). Long-Acting Injectable Antipsychotics: A Systematic Review of Their Non-Systemic Adverse Effect Profile. Neuropsychiatr. Dis. Treat..

[7] Su J.T., Simpson S.M., Sung S., Tfaily E.B., Veazey R., Marzinke M., Qiu J., Watrous D., Widanapathirana L., Pearson E. (2020). A Subcutaneous Implant of Tenofovir Alafenamide Fumarate Causes Local Inflammation and Tissue Necrosis in Rabbits and Macaques. Antimicrob. Agents Chemother..

[8] Shakil A., Hern F.Y., Liu C., Temburnikar K., Chambon P., Liptrott N., McDonald T.O., Neary M., Owen A., Freel Meyers C. (2022). Linear and branched polymer prodrugs of the water-soluble nucleoside reverse-transcriptase inhibitor emtricitabine as structural materials for long-acting implants. J. Mater. Chem. B.

[9] Liptrott N.J., Giardiello M., McDonald T.O., Rannard S.P., Owen A. (2018). Assessment of interactions of efavirenz solid drug nanoparticles with human immunological and haematological systems. J. Nanobiotechnol..

[10] Heaton B.J., Jensen R.L., Line J., David C.A.W., Brain D.E., Chadwick A.E., Liptrott N.J. (2022). Exposure of human immune cells, to the antiretrovirals efavirenz and lopinavir, leads to lower glucose uptake and altered bioenergetic cell profiles through interactions with SLC2A1. Biomed. Pharmacother..

[11] Tirumala M.G., Anchi P., Raja S., Rachamalla M., Godugu C. (2021). Novel Methods and Approaches for Safety Evaluation of Nanoparticle Formulations: A Focus Towards In Vitro Models and Adverse Outcome Pathways. Front. Pharmacol..

[12] Szebeni J. (2014). Complement activation-related pseudoallergy: A stress reaction in blood triggered by nanomedicines and biologicals. Mol. Immunol..

[13] Chanan-Khan A., Szebeni J., Savay S., Liebes L., Rafique N.M., Alving C.R., Muggia F.M. (2003). Complement activation following first exposure to pegylated liposomal doxorubicin (Doxil^®^): Possible role in hypersensitivity reactions. Ann. Oncol..

[14] Plant-Hately A.J., Eryilmaz B., David C.A.W., Brain D.E., Heaton B.J., Perrie Y., Liptrott N.J. (2022). Exposure of the Basophilic Cell Line KU812 to Liposomes Reveals Activation Profiles Associated with Potential Anaphylactic Responses Linked to Physico-Chemical Characteristics. Pharmaceutics.

[15] Hobson J.J., Al-khouja A., Curley P., Meyers D., Flexner C., Siccardi M., Owen A., Meyers C.F., Rannard S.P. (2019). Semi-solid prodrug nanoparticles for long-acting delivery of water-soluble antiretroviral drugs within combination HIV therapies. Nat. Commun..

[16] Liu C., Hern F.Y., Shakil A., Temburnikar K., Chambon P., Liptrott N., McDonald T.O., Neary M., Flexner C., Owen A. (2023). Polymer–prodrug conjugates as candidates for degradable, long-acting implants, releasing the water-soluble nucleoside reverse-transcriptase inhibitor emtricitabine. J. Mater. Chem. B.

[17] David C.A.W., Vermeulen J.P., Gioria S., Vandebriel R.J., Liptrott N.J. (2024). Nano(bio)Materials Do Not Affect Macrophage Phenotype—A Study Conducted by the REFINE Project. Int. J. Mol. Sci..

[18] Potter T.M., Cedrone E., Skoczen S.L., Neun B.W., Dobrovolskaia M.A. NCL Method ITA-6.1: Leukocyte Proliferation Assay. https://ncl.cancer.gov/resources/assay-cascade-protocols.

[19] HIV Drug Interactions. Emtricitabine PK Fact Sheet. http://www.hiv-druginteractions.org/prescribing_resources/hiv-pk-emtricitabine.

[20] Neun B.W., Ilinskaya A.N., Dobrovolskaia M.A., McNeil S.E. (2018). Analysis of Complement Activation by Nanoparticles. Characterization of Nanoparticles Intended for Drug Delivery.

[21] Cedrone E., Neun B.W., Rodriguez J., Vermilya A., Clogston J.D., McNeil S.E., Barenholz Y., Szebeni J., Dobrovolskaia M.A. (2017). Anticoagulants Influence the Performance of In Vitro Assays Intended for Characterization of Nanotechnology-Based Formulations. Molecules.

[22] David C.A.W., Barrow M., Murray P., Rosseinsky M.J., Owen A., Liptrott N.J. (2020). In Vitro Determination of the Immunogenic Impact of Nanomaterials on Primary Peripheral Blood Mononuclear Cells. Int. J. Mol. Sci..

